# Acknowledgement

**Published:** 1883-04

**Authors:** 


### ACKNOWLEDGEMENT.

The Columbia College Museum has been recently enriched by the donation of some very rare, interesting and valuable specimens. The donation was made by the nestor of the veterinary profession, Prof. Robert Jennings, of Detroit, Mich.

